# Statistical considerations in a systematic review of proxy measures of clinical behaviour

**DOI:** 10.1186/1748-5908-5-20

**Published:** 2010-02-26

**Authors:** Heather O Dickinson, Susan Hrisos, Martin P Eccles, Jill Francis, Marie Johnston

**Affiliations:** 1 Institute of Health and Society, Newcastle University, 21 Claremont Place, Newcastle upon Tyne, NE2 4AA, UK; 2 Health Services Research Unit, University of Aberdeen, Health Sciences Building, Foresterhill, Aberdeen, AB25 2ZD, UK; 3 Department of Psychology, University of Aberdeen, Health Sciences Building, Foresterhill, Aberdeen, AB25 2ZD, UK

## Abstract

**Background:**

Studies included in a related systematic review used a variety of statistical methods to summarise clinical behaviour and to compare proxy (or indirect) and direct (observed) methods of measuring it. The objective of the present review was to assess the validity of these statistical methods and make appropriate recommendations.

**Methods:**

Electronic bibliographic databases were searched to identify studies meeting specified inclusion criteria. Potentially relevant studies were screened for inclusion independently by two reviewers. This was followed by systematic abstraction and categorization of statistical methods, as well as critical assessment of these methods.

**Results:**

Fifteen reports (of 11 studies) met the inclusion criteria. Thirteen analysed individual clinical actions separately and presented a variety of summary statistics: sensitivity was available in eight reports and specificity in six, but four reports treated different actions interchangeably. Seven reports combined several actions into summary measures of behaviour: five reports compared means on direct and proxy measures using analysis of variance or t-tests; four reported the Pearson correlation; none compared direct and proxy measures over the range of their values. Four reports comparing individual items used appropriate statistical methods, but reports that compared summary scores did not.

**Conclusions:**

We recommend sensitivity and positive predictive value as statistics to assess agreement of direct and proxy measures of individual clinical actions. Summary measures should be reliable, repeatable, capture a single underlying aspect of behaviour, and map that construct onto a valid measurement scale. The relationship between the direct and proxy measures should be evaluated over the entire range of the direct measure and describe not only the mean of the proxy measure for any specific value of the direct measure, but also the range of variability of the proxy measure. The evidence about the relationship between direct and proxy methods of assessing clinical behaviour is weak.

## Background

Over the past 15 years, there has been a concerted move to encourage the practice of evidence-based medicine [1]. The implementation of evidence-based recommendations and clinical guidelines often needs changes in the behaviour of healthcare professionals. Evaluation of the effectiveness of initiatives to change clinical behaviour requires valid measures of such behaviour, which are relevant to policy-makers, practitioners, and researchers.

Clinical practice can be measured by direct observation, which is generally considered to provide an accurate reflection of the observed behaviour and therefore represent a 'gold standard' measure. However, direct measures can be intrusive and can alter the behaviour of the individuals being observed, placing significant limitations on their use in any other than small studies. As they are also time-consuming and costly, they are not always a feasible option. Measurement of clinical behaviour has therefore commonly relied on indirect measures, including review of medical records (or charts); clinician self-report, and patient report. However, the extent to which these proxy measures of clinical behaviour accurately reflect a clinician's actual behaviour is unclear. In a separate systematic review, we assessed the validity of proxy measures for directly observed clinical behaviour [2]. The included studies used a variety of statistical methods both to summarize clinical behaviour and to compare proxy and direct measures. The estimated agreement between direct and proxy measures varied considerably not only between different clinical actions but also between studies. It seems unlikely that all the methods used will have similar validity: some of the heterogeneity in findings may be due to inappropriate statistical methods. The planning of future studies would benefit from an evaluation of the range of approaches used. The objective of the present paper is to evaluate the validity of the statistical methods used by these studies and to recommend the most appropriate methods.

## Methods

In a companion systematic review [2], evidence was synthesised from empirical, quantitative studies that compared a measure of the behaviour of clinicians (doctors, nurses, and allied health professionals) based on direct observation (standardised patient, trained observer, or video/audio recording) with a proxy measure (retrospective self-report; patient-report; or chart-review) of the same behaviour. The review searched PsycINFO, MEDLINE, EMBASE, CINAHL, Cochrane Central Register of Controlled Trials, Science/Social science citation index, Current contents (social and behavioural med/clinical med), ISI conference proceedings, and Index to Theses for studies that met the inclusion criteria. All titles, abstracts, and full text articles retrieved by electronic searching were screened for inclusion, and data were abstracted independently by two reviewers. Disagreements were resolved by discussion with a third reviewer where necessary.

All the studies identified as meeting the inclusion criteria for the review based their measures of behaviour on whether a clinician had performed one or more clinical actions, *e.g.*, prescribing a specific drug, ordering a specific test, asking a patient whether s/he smoked. Hence, clinical actions were recorded as binary (yes/no) variables, which we refer to as 'items'. Several studies compared direct and proxy values of items, but others combined items into summary scores that were treated as continuous variables and then compared the summary scores based on direct and proxy measures. So, for the purposes of assessing the statistical methods, we divided the methods used into those that compared items and those that compared summary scores.

### Item by item comparisons

We noted whether studies reported the sensitivity, specificity, positive predictive value, or negative predictive value of the proxy measure (see Table 1); we noted any alternative methods used to summarise the relationship between direct and proxy measures.

**Table 1:** Statistics summarising validity of binary (yes/no) measures of behaviour

	Direct measure		
**Proxy measure:**	**YES**	**NO**	**TOTAL**
**YES**	a	b	a + b
**NO**	c	d	c + d
**TOTAL**	a + c	b + d	T = a + b + c + d

The sensitivity of the proxy measures is defined as: a/(a+c); its specificity is as: d/(b+d); its positive predictive value as a/(a+b); and its negative predictive value as d/(c+d).

### Comparisons of summary scores

A proxy measure of behaviour will not be a consistent surrogate for a direct measure of behaviour unless both the proxy and direct measures are valid. The companion review assessed the face and content validity and reliability of these measures [2]. Here, we assessed four aspects of the statistical validity of the measures.

### Bias and variability

We noted whether studies reported the average relationship between direct and proxy measures, described over the entire range of possible values of the measures, and the variability around the average relationship, *e.g.*, by a Bland and Altman plot [3-7] or a regression line, regressing the direct on the proxy measure, with a prediction interval [7].

For all studies (comparing both items and summary scores), we also assessed the following:

1. Estimation or hypothesis testing: We noted whether studies treated comparisons between direct and proxy measures largely as estimation or hypothesis testing; we assumed that reporting of p-values indicated the latter.

2. Confidence intervals and clustering: We noted whether studies reported confidence intervals on statistics summarising the relationship between direct and proxy measures, and allowed for clustering of consultations within clinicians.

## Results

Fifteen reports of eleven studies met the inclusion criteria [8-22]; three of these studies were reported in more than one publication [10,9,19,8,12,16], but these publications used different statistical methods to compare direct and proxy measures, so they are considered separately.

### Study designs

All included reports (except [13]) used identical checklists and scoring procedures to rate both direct and proxy measures of behaviour. The number of items per consultation considered by each report ranged from one [13] to 79 [19] (see Table 2). Thirteen reports compared the direct and proxy measures item by item [8-12,14,15,17-22]; seven reports combined the items into summary scores for direct and proxy measures, which were then compared [12-15,17,18]; three reports used both methods [14,15,18].

**Table 2:** Statistical methods used in the included papers to compare direct and proxy measures of behaviour

Report	n_i_	n_j_	n_k_	Statistics used	Notes
**Item-by-item comparisons: items treated as distinct**					

Flocke, 2004[9]	10	19	138		
Stange, 1998[19]	79	32	138		
Ward, 1996[20]	2	26	41	Sensitivity = a/(a + c)	
Wilson, 1994[21]	3	20	16		
Zuckerman, 1975[22]	15	17	3		

Stange, 1998[19]	79	32	138		
Ward, 1996[20]	2	26	41		
Wilson, 1994[21]	3	20	16	Specificity = d/(b + d)	
Zuckerman, 1975[22]	15	17	3		

Dresselhaus, 2000*[8] Gerbert, 1988[11] Pbert, 1999*[15] Rethans, 1987*[18] Wilson, 1994[21]	7 4 15 24 3	8 3 9 1 20	20 63 12 25 16	Agreement: comparison of: (i) (a + b)/T, and (ii) (a + c)/T	Agreement was assessed by comparing the proportion of recommended behaviours performed as measured by the direct and proxy measures. Three reports performed hypothesis tests, using analysis of variance [8], Cochran's Q-test [15], and McNemar's test [18].

Gerbert, 1988*[11] Pbert, 1999*[15] Stange, 1998[19]	4 15 79	3 9 32	63 12 138	kappa = 2(ad - bc)/{(a + c)(c + d) + (b + d)(a + b)}	All three reports used kappa-statistics to summarise agreement; two reports [11,15] also used them for hypothesis testing.

Gerbert, 1988[11]	4	3	63	Disagreement = (i) c/T (ii) b/T (iii) (b + c)/T	Disagreement was assessed as the proportion of items recorded as performed by one measure but not by the other.

**Item-by-item comparisons: items treated as interchangeable within categories of behaviour**					

Luck, 2000[12]	NR	8	20		
Page, 1980 [14]	16-17	1	30	Sensitivity = a/(a + c)	
Rethans, 1994[17]	25-36	3	35		
	
Luck, 2000[12] Page, 1980[14]	NR	8 1	20 30	Specificity = d/(b + d)	

Gerbert, 1986[10] Page, 1980[14]	20 16-17	3 1	63 30	Convergent validity = (a + d)/T	Convergent validity was assessed as the proportion of items showing agreement.

**Comparisons of summary scores for each consultation: summary scores were the number (or proportion) of recommended items performed**					

Luck, 2000*[12]	NR	8	20		**Analysis of variance **to compare means of scores on direct measure and proxy.
Pbert, 1999*[15]	15	9	12		
				Summary score:	
Rethans, 1987*[18]	24	1	25		**Paired t-tests **to compare means of scores on direct measure and proxy.
		
Pbert, 1999*[15]	15	9	12		**Pearson correlation **of the scores on direct measure and proxy.

**Comparisons of summary scores for each clinician: summary scores were the number (or proportion) of recommended items performed**					

O'Boyle, 2001[13]	1	NA	120		**Comparison of means **of scores on direct measure and proxy.
				Summary score:	
O'Boyle, 2001*[13]	1	NA	120		**Pearson correlation **of scores on direct measure and proxy.
Rethans, 1994*[17]	25-36	3	25		

**Comparisons of summary scores for each consultation: summary scores were weighted sums of the number of recommended items performed**					

Peabody, 2000*[16]	21	8	28		**Analysis of variance **to compare means of scores on direct measure and proxy.
				Summary score:	
Page, 1980*[14]	16-17	1	30		**Pearson correlation **of scores on direct measure and proxy.

a, b, c, d, T are defined in Table 1; i = item, j = consultation, k = physician, n_i _= average number of items per consultation, n_j _= average number of consultations per clinician; n_k _= average number of clinicians assessed; ω_i _= weight for i^th ^item; x_ijk _= 0 if item is not performed; x_ijk _= 1 if item is performed;.

NR = Not reported; NA = Not applicable.

* This study used this method for hypothesis testing.

### Reports comparing items

Seven reports [8,9,11,19-22] did not attempt to combine items in any way. Two reports [15,18] analysed items both as separate items and also after amalgamation into a summary score for each consultation (see below).

### Reports comparing items, but treating items as interchangeable within categories of behaviour

Four reports treated different items interchangeably within specific categories: necessary, unnecessary behaviours [12]; assessing symptoms, assessing signs, ordering laboratory tests, delivering treatments, delivering patient education [10]; must do, should do, could do, should not do, must not do actions [14]; taking a history, performing a physical examination, ordering laboratory examinations, giving guidance and advice, delivering medication and therapy, specifying follow-up [17].

### Reports combining items into summary scores for each consultation

Four reports constructed summary scores, essentially defined as the number of recommended items that were performed, for each consultation, using both the direct and proxy measures [12,14-16]. Two of these reports [14,16] weighted the items to reflect their perceived importance.

One further report constructed summary scores for each consultation by category of item: obligatory, intermediate, and superfluous [18]. This study had only one consultation/clinician, so its summary score could equally well be regarded as describing the clinician or the consultation.

### Reports combining items into summary scores for each clinician

Two reports constructed summary scores for each clinician, using both the direct and proxy measures. One report recorded only one item (hand washing) and constructed a summary score for each clinician by calculating the number of times the item was performed in a two-hour period as a proportion of the number of times it should have been performed [13]. The other report recorded a clinician's behaviour on several items in up to four consultations and constructed a summary score for each clinician by summing the number of recommended items performed in all consultations [17].

### Statistical methods used to compare direct and proxy measures

Table 1 summarises the statistical methods used in the included papers to compare direct and proxy measures of behaviour.

### Item by item comparisons

Six reports presented sensitivity [9,12,17,19-21], and a further two presented sufficient data to allow calculation of the sensitivity [14,22]. Three reports presented specificity [12,19,20], one report [21] presented the proportion of false positives (1-specificity); and two reports presented sufficient data to allow calculation of the specificity [14,22]. However, some of these reports treated items describing different clinical actions as interchangeable within broad categories of behaviour [12,14,17]. No reports presented the positive or negative predictive values.

Five reports presented agreement [8,11,15,18,21] based on the percentage of recommended behaviours performed as measured by the direct and proxy measures. Three of these reports tested the null hypothesis that these proportions were the same, using either analysis of variance [8], Cochran's Q-test [15] or McNemar's test [18]. Both Cochran's Q-test and McNemar's test evaluate the hypothesis that the proportions positive on the direct measure and proxy are the same but, unlike McNemar's test, Cochran's Q-test can be used for tables with more than two methods of measuring behaviour [23]

Three reports presented kappa-statistics [11,15,19] to summarise agreement; two of these reports [11,15] also used them to test the null hypothesis that there was no more agreement between the methods than would be expected by chance.

One report presented disagreement [11] measured as: the proportion of items recorded as performed by the direct measure but not by the proxy measure; the proportion of items recorded as not performed by the direct measure but recorded as performed by the proxy measure; and the total of these.

Two reports presented 'convergent validity' [10,14], defined as the total number of items showing agreement (either present/present or absent/absent) on the two measures, as a proportion of the total number of items recorded by either measure. Both reports treated items describing different clinical actions as interchangeable. One report [10] calculated the convergent validity separately for each of 20 items in each consultation, assigned items to one of five categories, and presented the median convergent validity within each category as well as overall; the other report [14] pooled items within five categories and then calculated the convergent validity.

Inter-rater reliability was reported in six of the thirteen studies that compared measures item-by-item [14,17-21]; it ranged from 0.39 to 1.0.

### Comparisons of summary scores

All seven reports that compared summary scores used hypothesis testing. Three reports used analysis of variance or t-tests to test the null hypothesis that the mean scores from direct and proxy measures were the same [12,16,18]; three reports used the Pearson correlation to test the hypothesis that the scores were not correlated [13,14,17]; one report used both methods [15].

None of the reports plotted the data to compare direct and proxy measures or used any other method of showing how the direct and proxy were related over the entire range of their values or the variability in their relationship.

Inter-rater reliability was reported in four of the seven studies that compared summary scores [13,14,17,18] it ranged from 0.76 to 1.0.

## Discussion

Based on a companion systematic review of proxy measures of clinical behaviour [2], we further reviewed the wide range of statistical methods used in the included studies to compare proxy and direct measures of behaviour. We now discuss these statistical methods and then go on to make recommendations. Although our review was not, in principle, limited to measures based on binary (yes/no) items, all included papers used this approach. Because some papers compared items directly, and others compared scores based on combining item responses, we structure our discussion to reflect these two approaches.

### Item-by-item comparisons

In the current context, sensitivity answers the question: What proportion of actions that were actually performed and recorded by direct observation were identified by the proxy? The positive predictive value answers the question: What proportion of actions that were flagged by the proxy as having been performed were recorded by direct observation as performed? Specificity and negative predictive values address similar questions, but about actions that were not performed.

For single item comparisons, reporting of sensitivity and specificity is an appropriate way to assess the performance of a proxy [9,19-22], although thought needs to be given to which of these measures is most relevant to the clinical context and the research question, or whether both measures are required, or whether the positive (and/or negative) predictive value may be more informative. The positive and negative predictive values have the disadvantage that they vary with the prevalence of actual behaviour and so will vary between populations [24].

However, it is doubtful whether it is appropriate to estimate sensitivities and specificities based on a combination of items describing different clinical actions [10,12,14,17]. For example, it seems questionable whether it is valid to combine actions to review drugs and to discuss smoking cessation [10], or actions to ask the patient about the radiation of pain and to ask their occupation [12], or actions to apply a sling and to refer to a physiotherapist [17]. Combining items assumes that their proxy measures have the same underlying sensitivity and specificity, which may not be true. The validity of this assumption could be assessed and items combined only if their sensitivities and specificities were similar.

Assessment of 'agreement' by comparison of the proportion of items performed that were identified by the direct measure and proxy [8,11,15,18,21] is inappropriate because, unlike the sensitivity, it gives no indication of whether an item recorded as performed on the direct measure is likewise recorded as performed on the proxy. It is possible to have perfect agreement even if the direct and proxy measures record completely different items as performed. For example, the percentages recorded as performed by a direct measure and by the proxy can both be 50%, even if the sensitivity, specificity, positive and negative predictive value are all zero (*e.g.*, if a = d = 0 and b = c = 50; see Table 1). Furthermore, assessment of 'agreement' treats the direct and proxy measures as having equal validity, which may not necessarily be the case as either measure may pose validity problems.

Some reports [11,15,19] used kappa-statistics to quantify levels of agreement between direct and proxy measures. Although it is sometimes claimed that the kappa-statistic gives a 'chance-corrected' measure of agreement between two measures, it has been argued that this is misleading because the measures are clearly not independent [25]. Two of these reports [11,15] also used kappa-statistics to test the hypothesis that there is no more agreement between direct and proxy measures than might occur by chance. This is not very informative, since the measures are dependent by definition because they are rating the same behaviour. Kappa-statistics also share the flaws of other measures of correlation (the Pearson correlation and the intra-class correlation) for assessing agreement between methods of measurement: they assume that the two methods to be compared are interchangeable, whereas we usually regard the direct measure as being closer to the true value than the proxy; and their value is influenced by the range of measurement, with a wider range giving a higher correlation [26].

The same criticisms apply to assessment of 'disagreement'[11]. The 'convergent validity' [10] assumes that not performing specific actions has the same importance as performing them, which may or may not be true depending on the situation.

None of the reports allowed for clustering of items within clinicians, for example by using a multi-level model [27]. It is likely that there will be correlation of items within clinicians as actions performed by one clinician are likely to be more similar to each other than to actions performed by other clinicians. Failure to allow for this lack of independence of items is likely to result in spuriously precise estimates of sensitivity, specificity, and other summary statistics. Unfortunately, none of these reports presented confidence intervals on any of the summary statistics.

### Recommended methods to compare direct and proxy measures item by item

Individual items may be assessed for face and content validity by a group of subject matter experts. Their reliability may be assessed using a random or systematic sample of clinicians selected from a regional or national sampling frame [2]. If the focus of interest is actions that were performed, then the sensitivity and positive predictive value are appropriate statistics for comparing direct and proxy measures item-by-item. The proxy measure should have a high sensitivity and a high positive predictive value, such that it detects most actions that were performed and most actions that it flags as performed were actually performed. If actions that were not performed are also of interest, then the specificity and negative predictive value are also required. Items that assess different actions should not be treated as if they were interchangeable, unless they have been shown to have similar diagnostic properties.

### Comparisons of summary scores

Individual items may function as either indicator variables or as causal variables [28,29]. Indicator variables are determined by an unobservable, underlying concept: for example, the responses to items in an intelligence test are assumed to be determined by an underlying level of ability, and so they are expected to be correlated. In contrast, causal variables jointly determine an unobserved construct. For example, socio-economic status may be determined jointly by education, income, neighbourhood, and occupational prestige; an increase in any of these might increase socio-economic status, but we would not expect these indicators to be correlated. The methods used to combine items into scores depend on whether the items are regarded as indicator variables or causal variables. Item response theory, including Rasch models, may be applied to indicator variables [30,31], but is inappropriate for causal variables, for which a range of methods have been proposed [28]. None of the included reports contained any discussion of whether the items were regarded as causal or indicator variables, although two reports [14,16] weighted items to reflect their importance.

Several reports compared the means of summary scores [12,13,15,16,18], which is inadequate for assessment of agreement. First, even if the means of the direct and proxy measures are similar, it cannot be assumed that they agree for all values of the direct measure. Second, the means do not give enough information to predict the direct measure from a value of the proxy. Third, comparison of means does not tell us anything about the variability of the proxy measure for any specific value of the direct measure. Finally, it is possible for summary scores to have the same value for direct measure and proxy measures, even if the responses to the individual items are very different.

Some reports calculated summary scores for each consultation [12-16,18], whereas other reports averaged the consultation scores for each clinician in order to obtain a score for the clinician [17]. Simply averaging over consultations does not allow for the correlation of actions by the same clinician (discussed above): methods such as multi-level modelling are required [27]. However, one report claimed, on the basis of analysis of variance, that there was no significant effect of clustering within clinicians [15].

Several reports used methods based on a linear model-analysis of variance [12,15,16], t-tests [18], or correlation [13-15,17]-to assess agreement. These methods assume that the outcome of interest is continuous and normally distributed. This is not strictly valid when the outcome is the proportion of items performed, as proportions have discrete values and a binomial distribution, although in many cases, the inferences that are made may still be valid.

Analysis of variance assesses how the mean value of a variable is affected by the classification of the data [23]. It compares the variation between groups (in this case, measurements by direct and proxy methods) with the variation within groups, in order to assess whether the mean values differ in different groups. Although this method has the advantage that it can allow for other factors which might affect differences between methods, *e.g.*, disease, case complexity, physician training level, and hospital sites, it is essentially a method of testing the hypothesis that means are the same in different groups, which is inappropriate for the reasons given below. T-tests are a special case of analysis of variance and share its disadvantages.

The Pearson correlation measures the strength of linear association between two variables, and therefore gives a measure of the average variability in their relationship [23]. If a scatter plot of the two variables shows that all the points lie on a straight line, the Pearson correlation has the value of one (or minus one); but if the points show a lot of scatter, the Pearson correlation has a value between zero and one (or minus one). However, it has the disadvantage that it does not assess bias: for example, two measures can have perfect correlation (equal to one) even if one measure is consistently twice the other measure [5,7]. Furthermore, the Pearson correlation depends on the range of the variables: if there is indeed a linear relationship between the variables, then a wider range of variation of behaviours will result in a higher correlation coefficient [3,5,7].

All of the reports that compared summary scores used hypothesis testing. Assessment of agreement between two measures is a problem of estimation, not hypothesis testing [3]. Estimation can predict the value that one measure (the direct measure) is likely to take, if the value of the other measure (the proxy) is known. Hypothesis testing aims to aid decision-making about whether the observed data provide evidence that a particular hypothesis (*e.g.*, that two values are the same) is unlikely to be true. Hypothesis testing and estimation may lead to different conclusions: for example, if there is a wide range of variability in each measure, hypothesis testing is likely to lead to a conclusion that the proxy and direct measure are similar, whereas estimation would tend to indicate that the proxy may be a poor predictor of the direct measure.

### Recommended methods to compare proxy and direct measure summary scores

Measures that summarise several items should be reliable, repeatable, capture a single underlying aspect of behaviour, and measure that construct using a valid measurement scale. Once such direct and proxy measures have been constructed, the relationship between them should be evaluated over their entire range, first by a simple plot of one measure against the other [4,5]. The next step will depend on whether the direct measure can be regarded as an error-free 'gold standard'. In the studies included in our review, inter-rater reliability was good for direct measures based on simulated patients [14,17,18], suggesting that these measures had little error, but direct measures based on audio or video recording were more prone to errors [2].

If we want to assess agreement between two methods of measurement, neither of which can be regarded as estimating the true value of the quantity measured, Bland and Altman have recommended that the difference between two measures should then be plotted against their mean. This allows visual assessment of both systematic bias and of variation [3-7].

Alternatively, if one measure can be regarded as error-free, and interest centres mainly on whether it shows a consistent, predictable relationship with the proxy measure, the problem is one of calibration rather than assessment of agreement [4,7]. This relationship can then be captured by use of regression [6]: the regression line captures the average relationship between the measures, and it is possible to construct a 95% prediction interval that shows, for each value of the proxy measure, the range within which the values of the direct measure for an individual clinician (or consultation) are likely to lie.

This use of regression has some intrinsic weaknesses. First, as the proxy is inevitably measured with some error, the relationship between the direct and proxy measures will almost certainly show regression to the mean [32,33], thus underestimating high values of the direct measure and overestimating low values. Second, regression assumes that the amount of variation in the proxy scores does not depend on the value of direct measure, which was not true in the studies included in this review. The summary scores used in included studies had a limited range, *e.g.*, 0 to100, so the variation in the proxy score tended to be smaller if the direct scores were closer to the extremes. This could lead to spurious precision in estimates of the regression line and its prediction interval. Such an effect would be more marked for scores based on fewer items or with larger standard deviations. Third, as noted above for analysis of variance and correlation, the assumption that the summary score is continuous and normally distributed is not valid. Finally, the relationship between direct and proxy measures may not be linear over their entire range: non-linearity can be assessed by inspection of the plot or, more formally, by testing the effect of adding a quadratic term to the regression. Alternatives to a regression approach include item response theory [31] (if it is assumed that the items are indicator variables) or multiplicative utility formulae or structural equation modelling (if it is assumed that the items are causal variables) [28,34].

## Conclusions

The fifteen reports analysed in this review used a variety of methods to construct direct and proxy measures of clinical behaviour and to compare them. Four reports of four studies that compared individual items [9,19-21] used appropriate statistical methods-sensitivity and specificity-to do so. However, the reports that combined items into summary scores focused on comparing means of these scores, whereas it would have been more informative to describe the average relationship between direct and proxy scores and the variability around that average over the entire range of the scores. The paucity of this evidence and the heterogeneity of clinical behaviours limit the conclusions that can be made about the relationship between direct and proxy methods of assessing clinical behaviour.

## Competing interests

MPE is Co-Editor in Chief of Implementation Science. All editorial decisions on this manuscript were made by Co-Editor in Chief Brian Mittman.

## Authors' contributions

All authors contributed to the conception and design of the study. HD drafted the manuscript. All authors read and approved the submitted draft. HD, ME, JF, and SH reviewed the articles and abstracted the data.
